# A Novel Promoter CpG-Based Signature for Long-Term Survival Prediction of Breast Cancer Patients

**DOI:** 10.3389/fonc.2020.579692

**Published:** 2020-10-20

**Authors:** Yang Guo, Xiaoyun Mao, Zhen Qiao, Bo Chen, Feng Jin

**Affiliations:** Department of Breast Surgery, The First Affiliated Hospital of China Medical University, Shenyang, China

**Keywords:** DNA methylation, breast cancer, prognosis, overall-survival, signature

## Abstract

DNA methylation has been reported as one of the most critical epigenetic aberrations during the tumorigenesis and development of breast cancer (BC). This study explored a novel promoter CpG-based signature for long-term survival prediction of BC patients. We used The Cancer Genome Atlas (TCGA) data as training set, and results were validated in an independent dataset from Gene Expression Omnibus (GEO). First, the differential methylation CpG sites were screened in TCGA dataset, of which the candidate promoter CpG sites were preliminarily identified with the univariate Cox regression analysis and the least absolute shrinkage and selection operator regression analysis. Second, the signature was constructed with stepwise regression analysis and multivariate Cox proportional hazards model, which was validated with the survival analysis of two cohorts each from TCGA and GEO databases. The 10-year receiver operating characteristic curves of risk score presented an area under the curve of over 0.7 for both cohorts. A nomogram was also constructed and released. Moreover, Gene Set Enrichment Analysis was performed to identify the more active pathways in high-risk patients. The CpG sites–target gene correlations and differential methylation regions were further explored. In conclusion, the promoter CpG-based signature exhibited good prognostic prediction efficacy in the long-term overall survival of BC patients.

## Introduction

Breast cancer (BC) has become one of the most concerned public health issues in the worldwide, because of the growing incidence, high mortality, and huge economic burden (1, 2). More than 1 million new BC cases were diagnosed in 2002 (3). For women, BC led to <25% of the newly diagnosed cancer cases and caused 14.7% of cancer-associated deaths (4). Further, the treatment costs of BC have been generally escalated with the advance of disease stage at diagnosis (5). BC patients can greatly benefit from early diagnosis, both in therapeutic efficacy and economic burden.

With the advances of molecular diagnosis technology, the heterogeneity and complexity of BC have been revealed (6). BC can be classified into different subgroups based on histopathologic characteristics or gene expression profiles. The molecular characterization of BC would provide much information for understanding the pathogenesis of BC and exploring potential markers for early diagnosis and target therapy (7).

DNA methylation, mainly occurring at cytosine-phosphate-guanosine dinucleotide (also designated as CpG) where cytosine was converted to 5-methylcytosine (5meC), has been considered to make important effects in cancer development (8). The covalent addition of a methyl group was generally observed in cytosine within CpG dinucleotides, which were concentrated in large clusters called CpG islands (9). The abnormal methylation of promoter CpG dinucleotide sites in cancer leads to transcriptional silencing, which would be heritable in progeny cells (10). Because the DNA methylation can be varied with internal and external factors, it has become a research hotspot for investigating the tumorigenesis and cancer development (11). At the same time, DNA methylation has also been intensively explored as a target for epigenetic treatment.

Aberrant DNA methylation makes effects in BC. One study investigated the link between DNA methylation and gene expression in BC. The result identified a transcriptional network regulated by DNA methylation at enhancers, in a cell lineage–specific manner (12). Some BC-associated heritable DNA methylation markers were screened through detecting and comparing DNA methylation levels in BC patients and their family members (13). The endogenous and external factors may make effects via modulating DNA methylation patterns, such as oxidative DNA damage and age-related reproductive factors (14, 15).

After verifying the association between DNA methylation and gene expression in BC, some large-scale studies have been performed to comprehensively explore the potential DNA methylation markers with clinical significance (16). In another study, blood-based DNA methylation biomarkers were summarized, which showed significance in risk stratification or the early detection of BC (17).

Infinium HumanMethylation450 Bead Chip contained a total of 485,764 CpG dinucleotide sites in the human genome. The whole genome could be divided into four regions: promoter, body, 3′-UTR (3′-untranslated region) and intergenic region. The promoter region was subdivided into 5′-UTR (5′-untranslated region), TSS200 (within 200 bp upstream of the transcription start site), TSS1500 (within 1,500 bp upstream of the transcription start site), and 1stExon. According to the distance from CpG islands, CpG shores referred to the area within 2 kb upstream and downstream of CpG islands; CpG shelves referred to the area within 2 kb upstream and downstream of CpG shores; open sea referred to other areas except CpG islands, CpG shores, and CpG shelves. From the functional genome distribution standpoint, 200,339 CpG sites were located in promoter regions; 15,383, 150,212 and 119,830 CpG sites correspond to 3′-UTR, gene body, and intergenic–open sea sequences, respectively. From the CpG content and neighborhood context, 150,254 were located in CpG islands, 112,072 in CpG shores, 47,161 in CpG shelves, and 176,277 were isolated CpG sites in the genome defined as “open sea” (18).

In this study, we aimed to explore novel survival-associated promoter CpG dinucleotide sites for prognostic prediction in BC patients. The BC methylation 450 K datasets and corresponding clinical features were obtained from both The Cancer Genome Atlas (TCGA) and Gene Expression Omnibus (GEO) databases. First, the differential methylation CpG (dmCpG) sites were screened in TCGA dataset, of which the candidate promoter CpG sites were preliminarily identified with the univariate Cox regression analysis and the least absolute shrinkage and selection operator (LASSO) regression analysis. Second, the promoter CpG-based signature was constructed with stepwise regression analysis, which was validated with the survival analysis of two cohorts each from TCGA and GEO databases. A nomogram was also constructed, and the calibration curves showed that it was able to predict 5-, 7-, and 10-year survival accurately. Moreover, Gene Set Enrichment Analysis (GSEA) was performed to identify the more active pathways in high-risk patients and the CpG sites–target gene correlations and differential methylation regions (DMRs) were further explored.

## Materials and Methods

### Data Resources

Two datasets were included in this analysis. Breast-invasive carcinoma methylation 450 K dataset (designated as TCGA-BRCA) and clinical information of patients were downloaded from UCSC Xena database (https://xena.ucsc.edu/), involving 96 normal samples and 794 tumor samples. The dataset GSE72308 and corresponding clinical information were downloaded from the GEO database (designated as GEO-BRCA), including 295 patients (19). All the data were obtained based on the platform of Illumina Infinium Human Methylation 450 Bead Chip.

### Data Filter and Normalization

For the two datasets, the methylation beta matrix was filtered and normalized with ChAMP R package (20). The batch effect was removed with SVA R package. Probes in two datasets were filtered with the exclusion criteria including: (i) detection *p* > 0.01; (ii) bead count <3 in at least 5% of samples; (iii) non-CpG sites; (iv) all SNP-related probes and multihit probes; and (v) probes on X and Y chromosomes. The BMIQ method was applied for types I and II probe correction. In particular, probes with SNPs were identified in general 450 K SNP list (21) and filtered by champ.filter() function included in ChAMP R package.

### dmCpG Sites

The dmCpG sites were preliminarily screened with ChAMP R package. In this study, CpG sites with |Δβ| > 0.2 and Benjamini–Hochberg adjusted *P* < 0.05 were identified as dmCpG sites. The dmCpG sites located in the promoter regions (5′-UTR, TSS200, TSS1500 and 1stExon) were further screened and visualized with the pheatmap R package.

### The dmCpG Sites Identification in TCGA Dataset

TCGA dataset was applied as training cohort, of which only the patients with overall survival (OS) ranging from 90 to 3,650 days were included for further analysis. The males and patients without OS information were removed, and 715 patients were finally included. The univariate Cox regression analysis was applied for screening OS-associated dmCpG sites preliminarily with survival R package. The hazard ratio (HR) and *p*-value were provided. The dmCpG sites with *P* < 0.001 and HR < 10 were screened. A total of 78 survival-associated dmCpG sites were obtained, corresponding to 63 genes. The metascape (www.metascape.org) was applied for Gene Ontology (GO) function enrichment analysis (22). LASSO regression analysis was further performed to explore the key dmCpG sites with glmnet R package. Finally, 25 dmCpG sites were identified.

### The Construction of CpG-Based Prognostic Signature

With the identified 25 OS-associated dmCpG sites, stepwise regression analysis was applied to optimize the model. Variables with *P* < 0.01 in the stepwise analysis were included in a multivariate Cox proportional hazards model. Under the optimal situations, the dmCpG sites and corresponding coefficients were presented, and the formula for calculating the prognostic index (designated as risk score) based on methylation levels of dmCpG sites was obtained.

### The Relationship of Risk Score With Clinical Characteristics

A total of 565 patients with complete clinical information in TCGA dataset were included. The ggpubr R package and *t* test were involved to explore the relationships between the CpG-based risk score and clinical characteristics, including age and TNM (tumor, node, metastasis). *P* < 0.05 indicates statistically significant. The results were provided with boxplots.

### Independent Prognostic Prediction Analysis

Univariate and multivariate independent prognostic analyses were applied with survival R package. The included clinical variables were age, TNM (tumor, node, metastasis), and risk score. The 565 patients with complete clinical information in the TCGA dataset were included. The HR was calculated and expressed with forest plot. The 5-, 7-, and 10-year receiver operating characteristic (ROC) curves of risk score, age, and TNM (tumor, node, metastasis) were plotted with survival ROC R package. Area under the curve (AUC) of the ROC curve was also provided.

### The Validation of dmCpG-Based Signature

The prognostic prediction efficacy of dmCpG-based signature was validated in both TCGA and GEO datasets. The survival analysis was performed on 715 patients in TCGA dataset and 268 patients in GEO dataset, respectively, with survival R package. The risk score was calculated based on the methylation level of included dmCpG sites and corresponding coefficients. The patients could be classified as high- and low-risk groups, with the median risk score in TCGA dataset as cutoff value. The survival analyses of patients in high- and low-risk groups were performed with survival R package. The 5-, 7-, and 10-year ROC curves were plotted, and AUC of the ROC curve was calculated. Further, the nomogram was constructed for the patients in TCGA dataset with rms R package, and the calibration curves were also plotted with calibrate function.

### Analysis of Included dmCpG Sites

The dmCpG sites were further investigated. First, the correlation between dmCpG sites and located gene expression was explored. The RNA-Seq expression profile (FPKM) of TCGA-BRCA was downloaded from GDC Data Portal (https://portal.gdc.cancer.gov). The Pearson correlation coefficient between β value of dmCpG sites and FPKM value of located genes was calculated with cor.test in R and visualized. Second, based on the transcriptome data, the GSEA was performed to identify the more active pathways in low-risk patients with GSEA 4.0.1 with 184 background gene sets in c2.cp.kegg.v6.2.symbols.gmt. Finally, the DMRs were screened with ChAMP R package and annotated with wANNOVAR (http://wannovar.wglab.org/) (23). The KEGG pathway enrichment analysis was further performed with ConsensusPathDB (http://cpdb.molgen.mpg.de/) (24).

## Results

### The dmCpG Sites Identification

**Scheme 1:**
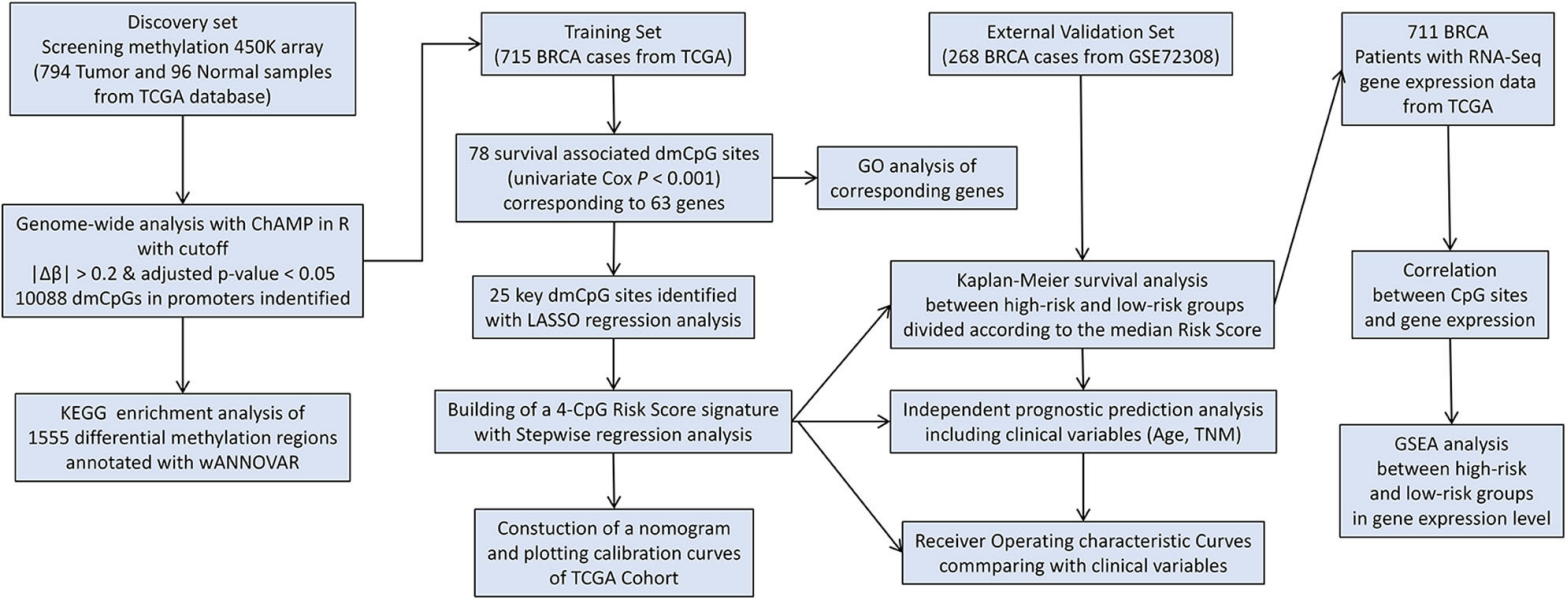
Flow diagram of the analysis procedure: data collection, processing, analysis, and validation.

**Figure 1:**
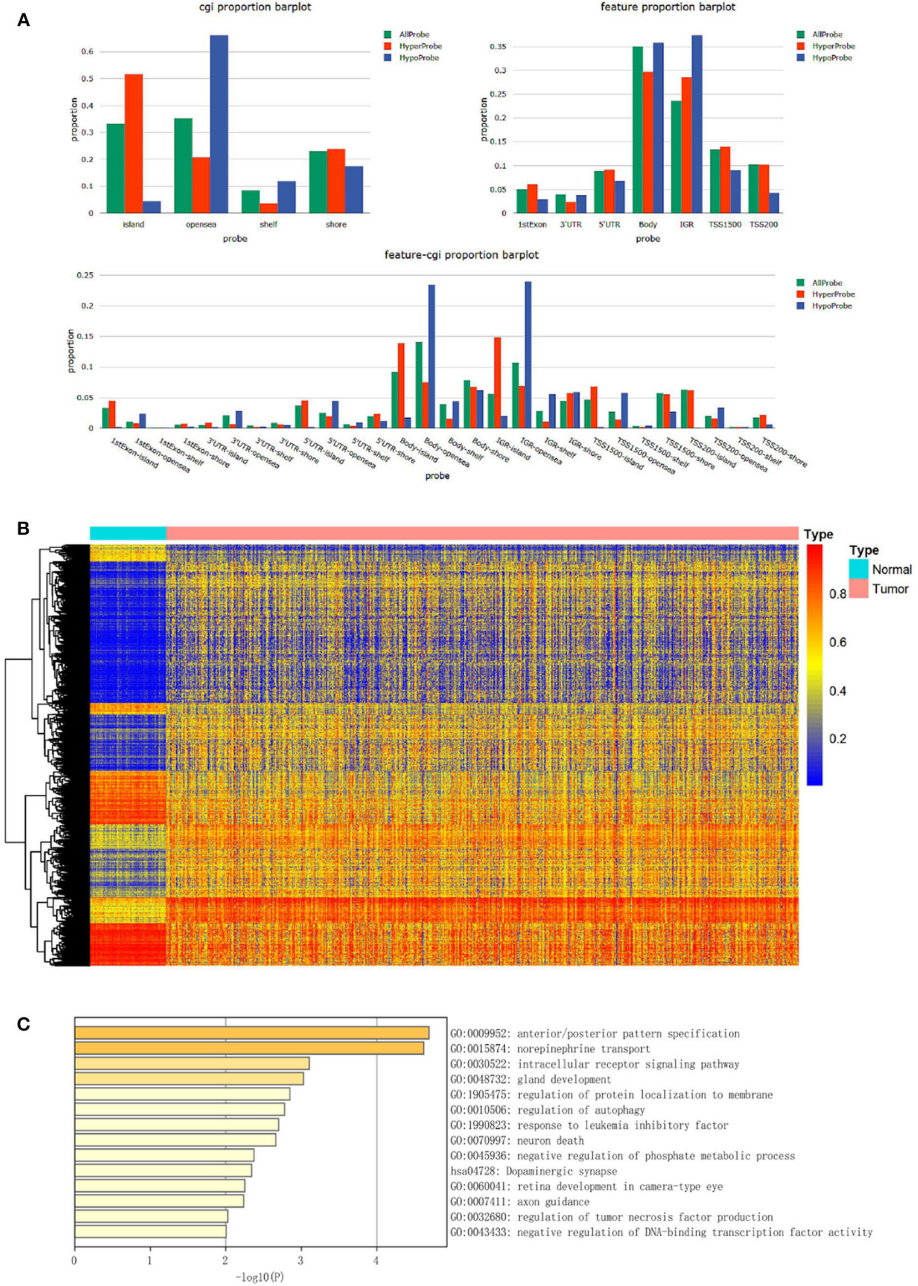
Identification and function enrichment of dmCpG sites in the TCGA cohort. **(A)** Histogram of the distribution regions of dmCpG sites in the genome. **(B)** Heatmap of dmCpG sites in the promoter region between normal and tumor samples. **(C)** GO function enrichment analysis of 78 dmCpG sites.

The study flowchart describing the process is shown in Scheme 1. The datasets from TCGA and GEO databases were preprocessed for further comparison, including filter, normalization, and batch correction. Then the dmCpG sites were screened in TCGA dataset with cutoff of |Δβ| > 0.2 and adjusted *P* < 0.05. The proportions of dmCpG sites were screened (Figure 1A), and a total of 10,088 dmCpG sites located in the promoter regions (5′-UTR, TSS200, TSS1500, and 1stExon) were finally identified (Figure 1B).

**Figure 2:**
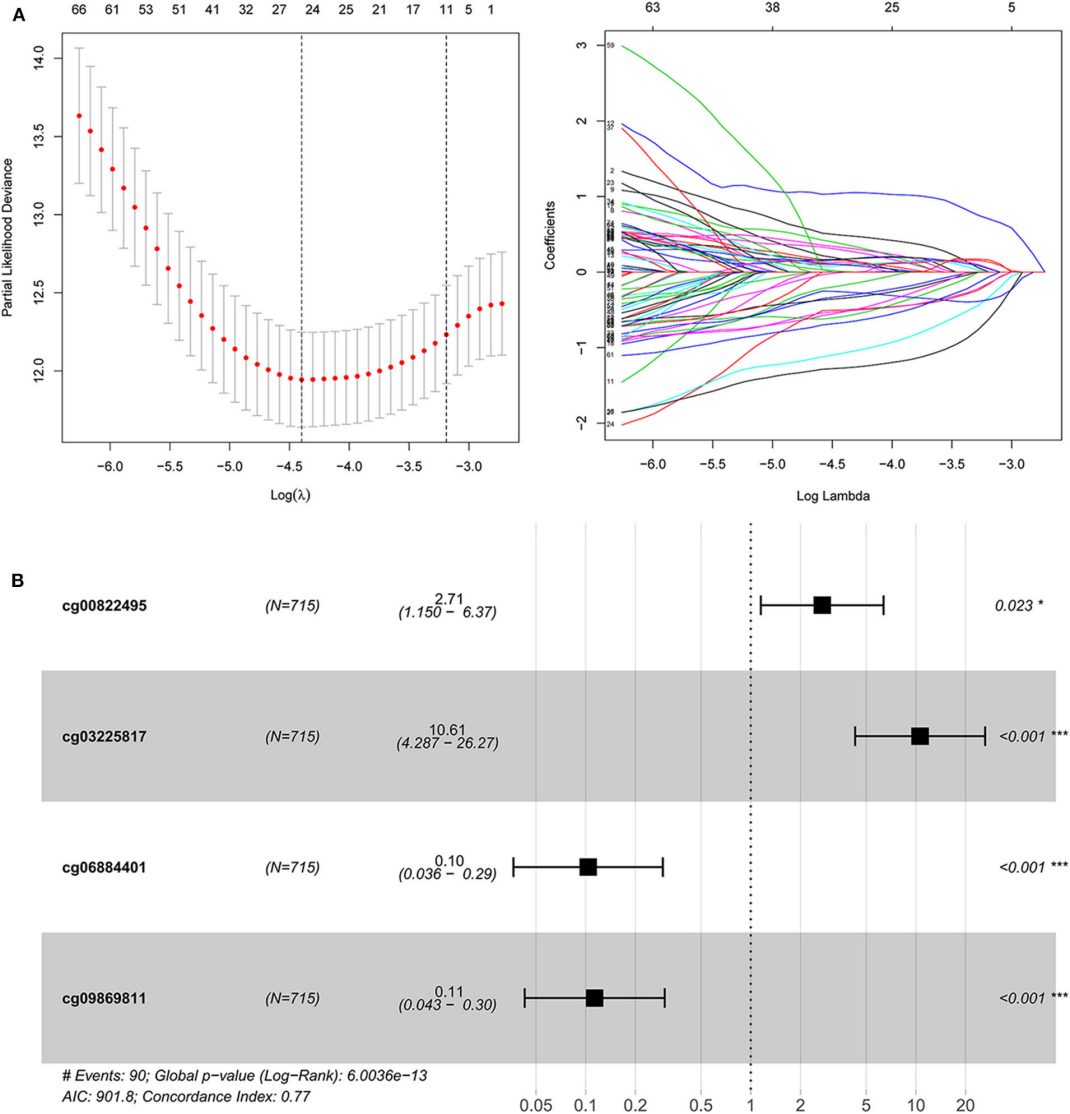
The construction of dmCpG-based prognostic signature. **(A)** LASSO regression analysis of CpG sites in the regions of promoter, with the selection of tuning parameter (lambda) and dynamic LASSO coefficient profiling. **(B)** The HR and coefficient of each dmCpG involved in the multivariate Cox proportional hazards model.

Second, the univariate Cox regression analysis was performed to identify the candidate OS-associated dmCpG sites in TCGA cohort. A total of 78 survival-associated dmCpG sites with *P* < 0.001 were identified, corresponding to 63 genes (Table 1). The GO function enrichment of the 63 genes indicated several GO terms, including anterior/posterior pattern specification, norepinephrine transport, intracellular receptor signaling pathway, gland development, regulation of autophagy, etc. (Figure 1C). LASSO regression analysis was further performed to explore the key dmCpG sites. Finally, 25 sites were identified (Figure 2A).

**Table 1:** The univariate Cox regression analysis of TCGA cohort.

**CpG ID**	**HR**	**HR.95L**	**HR.95H**	***p***	**Gene**	**CpG ID**	**HR**	**HR.95L**	**HR.95H**	***p***	**Gene**
cg03225817	9.135	3.827	21.807	0.000	*GRIA4*	cg17253709	0.031	0.005	0.214	0.000	*B3GNT2*
cg07972135	9.093	3.774	21.909	0.000	*GRIA4*	cg13525197	6.825	2.349	19.830	0.000	*ZSCAN23*
cg25482786	9.768	3.481	27.410	0.000	*CHL1*	cg01321962	0.274	0.134	0.563	0.000	*ESR1*
cg09093993	0.123	0.047	0.319	0.000	*PLEKHF2*	cg18784113	0.143	0.049	0.424	0.000	*THRB*
cg09869811	0.115	0.042	0.317	0.000	*NUTF2*	cg15379185	5.720	2.159	15.150	0.000	*EVX1*
cg03940684	5.322	2.397	11.817	0.000	*CHL1*	cg03329976	8.503	2.568	28.162	0.000	*SOX17*
cg07746943	6.081	2.570	14.392	0.000	*CHL1*	cg24970620	0.166	0.061	0.454	0.000	*SLC7A7*
cg06332621	0.110	0.038	0.325	0.000	*RBM47*	cg19002907	0.187	0.073	0.478	0.000	*TDRD1*
cg21217024	4.660	2.169	10.011	0.000	*GRIA4*	cg11429283	0.208	0.086	0.501	0.000	*SYTL2*
cg02631468	8.651	2.959	25.291	0.000	*VSX1*	cg04975519	0.189	0.074	0.481	0.000	*RNF32*
cg06818710	6.783	2.596	17.727	0.000	*ZSCAN23*	cg20924470	4.494	1.930	10.465	0.000	*FBXL21*
cg00043788	8.475	2.896	24.800	0.000	*VSX1*	cg02006107	0.164	0.059	0.454	0.001	*MRPS28*
cg17576603	0.155	0.060	0.398	0.000	*DAB2*	cg16703956	6.069	2.187	16.842	0.001	*SLC6A3*
cg01279654	0.193	0.084	0.444	0.000	*VGLL4*	cg27626299	7.201	2.347	22.088	0.001	*EVX1*
cg02919712	0.109	0.035	0.338	0.000	*DEPDC6*	cg10898212	0.167	0.061	0.463	0.001	*CDK5R1*
cg23559689	4.498	2.075	9.749	0.000	*GRIA4*	cg05144884	0.210	0.086	0.511	0.001	*PRSS27*
cg25609507	5.104	2.203	11.822	0.000	*CHAT*	cg14763548	3.816	1.780	8.184	0.001	*VSX1*
cg25160286	7.081	2.578	19.454	0.000	*EVX1*	cg09529093	0.215	0.089	0.519	0.001	*ANGPT1*
cg15287850	0.089	0.025	0.310	0.000	*ST6GAL1*	cg26065909	0.111	0.031	0.391	0.001	*SELENBP1*
cg09884146	0.047	0.010	0.230	0.000	*SPRED2*	cg04034767	3.489	1.703	7.151	0.001	*GRASP*
cg25520146	0.190	0.080	0.452	0.000	*HPYR1*	cg06327814	0.159	0.055	0.457	0.001	*C7orf53*
cg04054313	6.529	2.410	17.687	0.000	*GLT1D1*	cg13027458	0.181	0.068	0.483	0.001	*LOC644649*
cg10546487	0.115	0.036	0.365	0.000	*CLIC4*	cg12042659	3.690	1.738	7.831	0.001	*ZNF132*
cg05481991	5.512	2.217	13.704	0.000	*HMGA2*	cg08506163	6.358	2.187	18.485	0.001	*C6orf174*
cg08539965	0.105	0.032	0.350	0.000	*EIF4G3*	cg02337653	5.839	2.108	16.175	0.001	*C6orf174*
cg09968620	6.839	2.445	19.128	0.000	*HOXD9*	cg05223720	4.847	1.943	12.091	0.001	*CALN1*
cg10603275	4.181	1.943	8.997	0.000	*CHL1*	cg05863502	9.087	2.519	32.783	0.001	*CACNA1B*
cg00822495	4.572	2.020	10.349	0.000	*OTX2*	cg11523712	5.754	2.073	15.973	0.001	*HOXD13*
cg09260773	6.071	2.303	16.005	0.000	*TBX15*	cg27304204	0.240	0.104	0.555	0.001	*TSPAN8*
cg05663573	6.106	2.297	16.237	0.000	*SLC7A14*	cg26657920	0.203	0.079	0.517	0.001	*ACTBL2*
cg09871043	0.065	0.015	0.285	0.000	*PKHD1*	cg16636226	0.239	0.103	0.553	0.001	*LRRC3B*
cg13557668	5.153	2.117	12.545	0.000	*FBXL21*	cg14768785	4.648	1.886	11.453	0.001	*GHSR*
cg06884401	0.121	0.039	0.381	0.000	*FAM13A*	cg17465304	0.126	0.038	0.426	0.001	*KIF12*
cg16716750	4.143	1.915	8.963	0.000	*RGS17*	cg24475782	5.650	2.032	15.705	0.001	*TBX15*
cg23458558	5.638	2.190	14.516	0.000	*RALYL*	cg06578434	0.146	0.047	0.455	0.001	*SBNO2*
cg07277549	0.176	0.068	0.459	0.000	*NOD1*	cg14650610	3.463	1.661	7.222	0.001	*SPOCK1*
cg11413039	6.424	2.301	17.934	0.000	*PUS3*	cg00930873	5.258	1.969	14.046	0.001	*ALDH1A2*
cg18397523	6.636	2.331	18.887	0.000	*EVX1*	cg00880452	5.975	2.071	17.244	0.001	*SYCN*
cg01035160	4.861	2.027	11.659	0.000	*SNCA*	cg13032463	4.145	1.782	9.640	0.001	*T*

### The Construction of dmCpG-Based Prognostic Signature

With the identified 25 survival-associated dmCpG sites, the stepwise regression analysis was further applied to screen the candidate dmCpG sites for constructing the prognostic signature (Figure 2B). Ten candidate dmCpGs sites were screened in the stepwise analysis (Supplementary Figure 1). Further, four variables with *P* < 0.01 in the stepwise analysis were included in a multivariate Cox proportional hazards model. These four prognostic dmCpG sites were finally included in the model for calculating the risk score. In the optimal model, the AIC was 901.8, and concordance index was 0.77. The HR and coefficient of each dmCpG was provided (Figure 2C). The risk score was calculated with the following formula: risk score = cg00822495 × 0.9960 + cg03225817 × 2.3621 – cg06884401 × 2.2685 – cg09869811 × 2.1771.

### The Relationship of Risk Score With Clinical Characteristics

**Figure 3:**
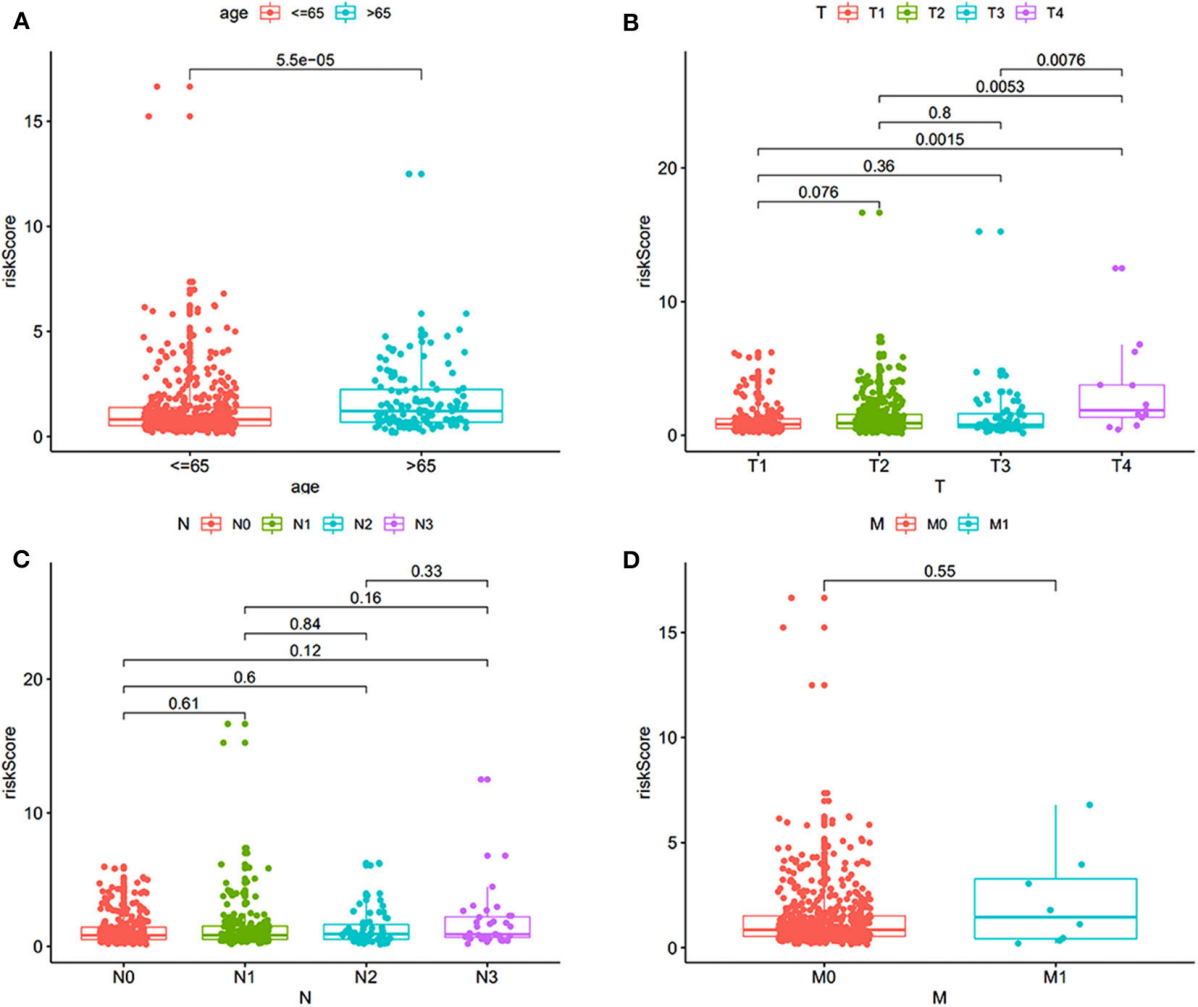
The correlation of risk score and clinical features, including age and TNM (tumor, node, metastasis). **(A)** Correlation with age. **(B)** Correlation with T stage. **(C)** Correlation with different lymph node status. **(D)** Correlation with distant metastasis.

A total of 565 patients with complete clinical information in TCGA dataset were included. The risk score was significantly higher in patients with older than 65 years (Figure 3A). Further, the risk score was significantly higher for patients at T4 compared to that of other T stages (Figure 3B). No differences were found in patients with different lymph node statuses and distant metastases (Figures 3C–D).

### Independent Prognostic Prediction Analysis

**Figure 4:**
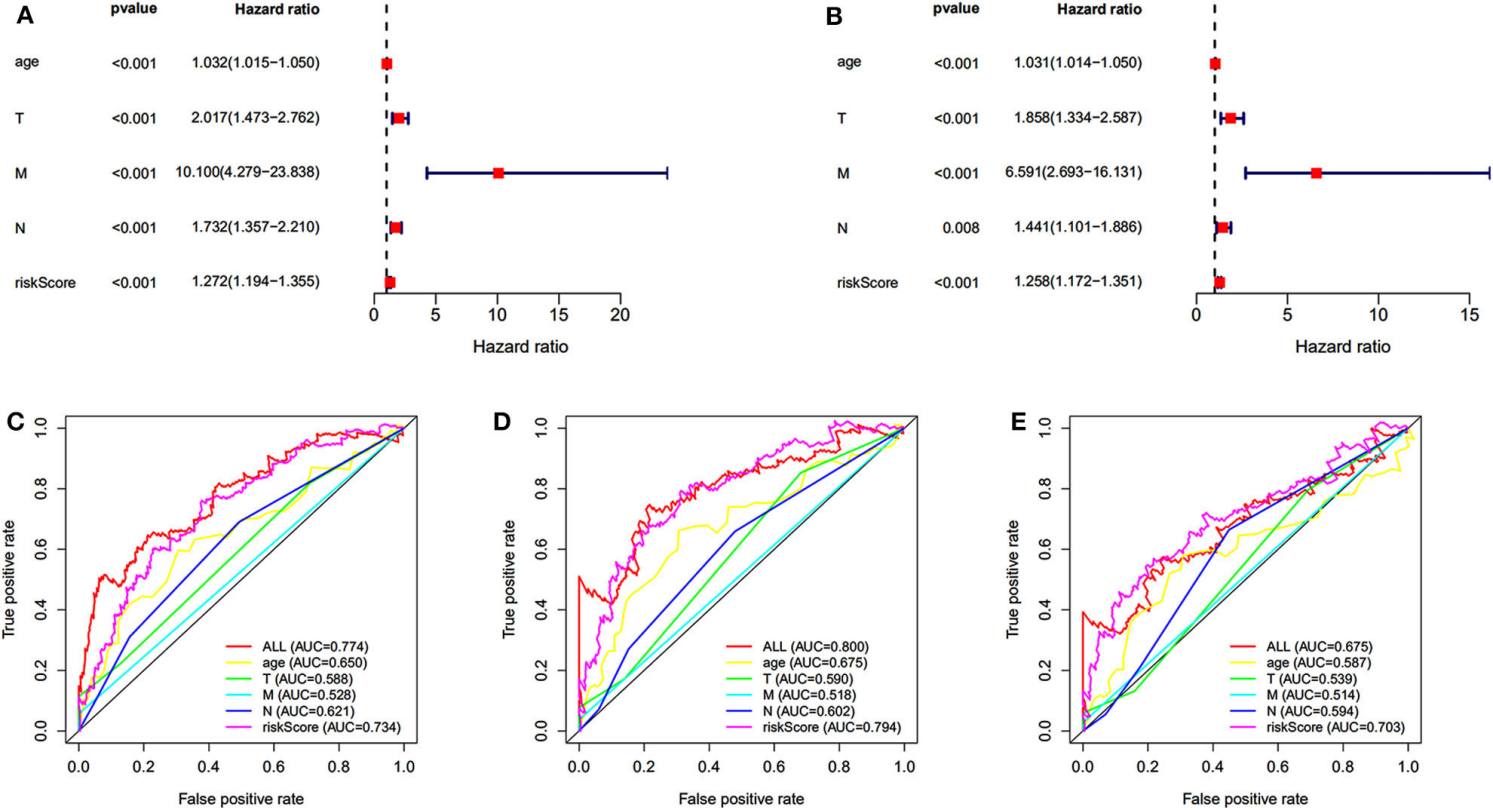
Independent prognostic prediction analysis. Univariate **(A)** and multivariate **(B)** survival analyses. **(C–E)** The 5-, 7-, and 10-year ROC curves of risk score, age, TNM (tumor, node, metastasis).

Univariate and multivariate survival analyses were performed to evaluate whether the risk score was an independent prognostic index irrespective of other clinical features. The clinical information of 565 patients in TCGA dataset was included. The univariate and multivariate analyses indicated that the age, TNM (tumor, node, metastasis), and risk score were an independent prognostic index (*P* < 0.05 for all, Figures 4A,B). The AUC of 5-, 7-, and 10-year ROC curves indicated that the risk score provided a higher value of AUC compared to that of other clinical features (Figures 4C–E). The risk score was verified as independent prognostic predictor. Further, the integration of risk score and clinical features provided similar AUC of 5-, 7-, and 10-year ROC curves compared to that of individual risk score, indicating comparable prognostic prediction efficacy.

### Prognostic Prediction Analysis

**Figure 5:**
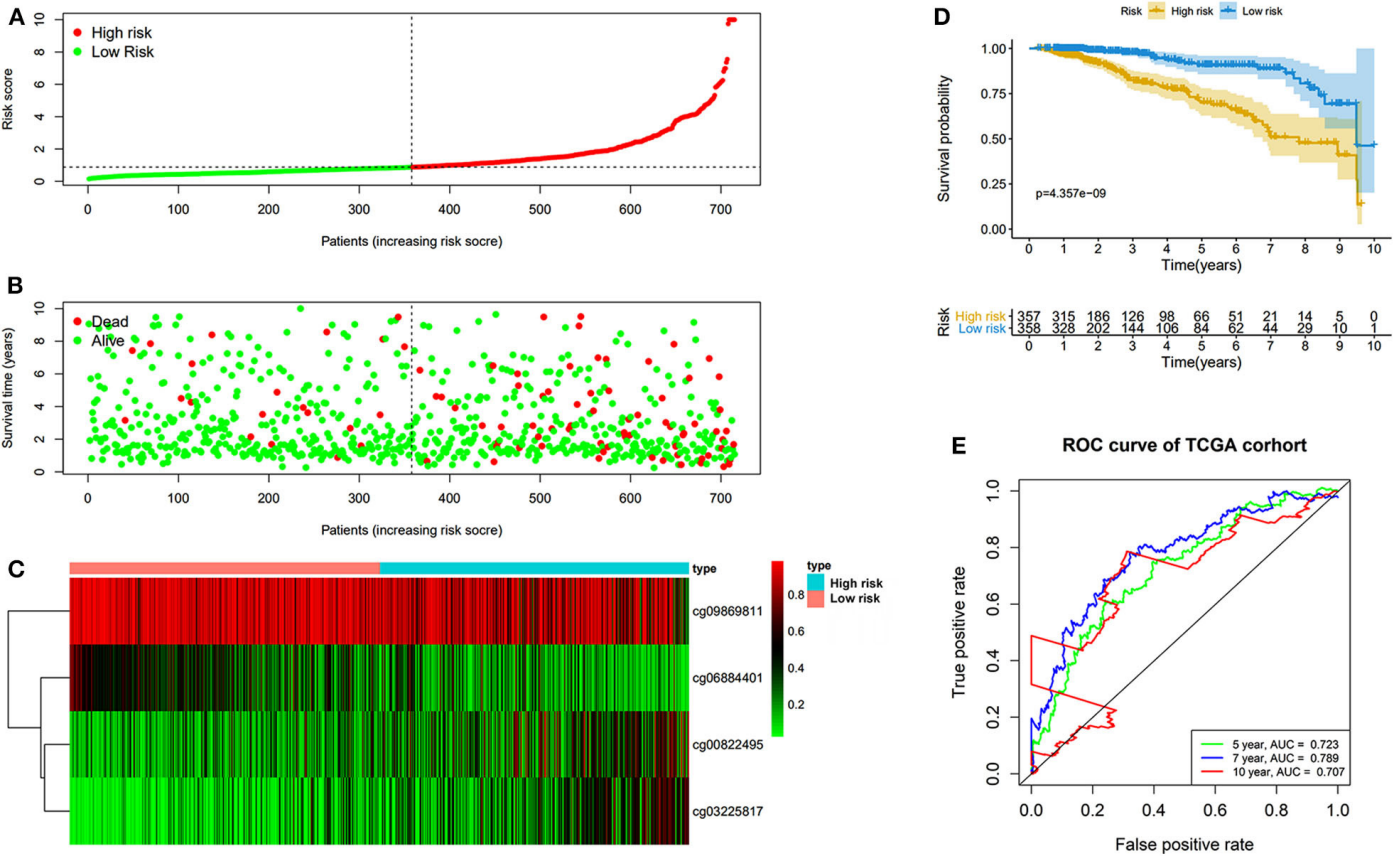
Risk score in the TCGA cohorts. **(A)** The rank of calculated risk score. **(B)** The survival status of high- and low-risk patients. **(C)** Heatmap of methylation level of 4 CpG sites. **(D)** Kaplan–Meier survival curve of the patients classified in high- and low-risk groups. **(E)** The 5-, 7-, and 10-year ROC curves of risk score.

**Figure 6:**
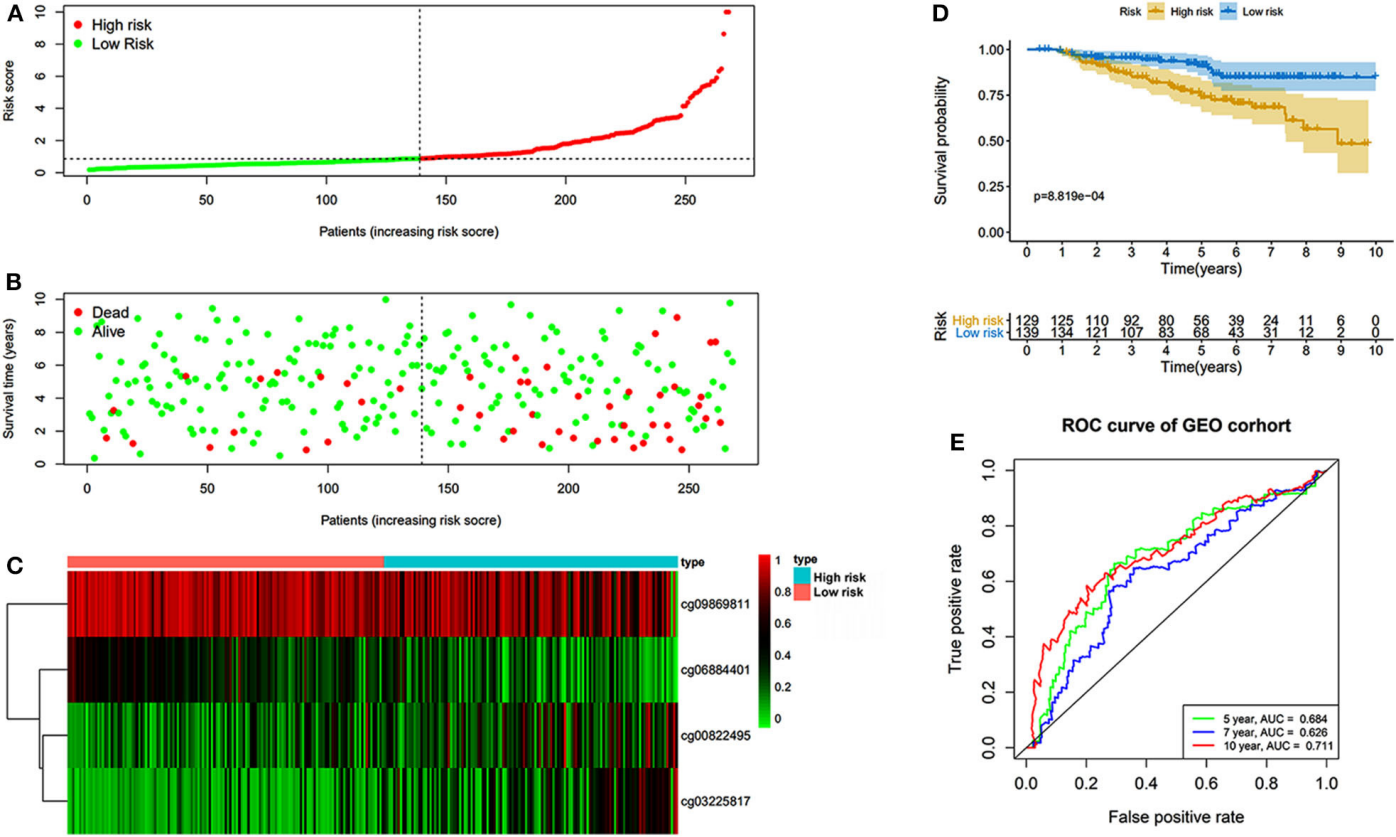
Risk score in the GEO cohorts. **(A)** The rank of calculated risk score. **(B)** The survival status of high- and low-risk patients. **(C)** Heatmap of methylation level of 4 CpG sites. **(D)** Kaplan–Meier survival curve of the patients classified in high- and low-risk groups. **(E)** The 5-, 7-, and 10-year ROC curves of risk score.

The prognostic prediction analysis was performed in TCGA and GEO cohorts, respectively. For patients in the TCGA cohort, risk score was calculated according to the normalized methylation levels of four dmCpG sites. The median risk score of 0.8775 was applied as the cutoff for dividing patients into high- and low-risk groups (Figure 5A). The distribution of survival status of all patients was also presented (Figure 5B). The variation tendency of the methylation level of dmCpG sites in heatmap was consistent with their coefficients in prognostic signature (Figure 5C). Kaplan–Meier survival analysis of the risk score indicated the survival probability of patients in high- and low-risk groups (*P* < 0.001, Figure 5D). Further, 5-, 7-, and 10-year ROC curves of risk score were plotted, with the AUCs of 0.723, 0.789, and 0.707, respectively (Figure 5E). The AUC value indicated good prognostic prediction efficacy. With the risk score of 0.8775 as cutoff, the prognostic prediction ability of risk score was also validated in the patients in the GEO cohort; similar results can be obtained (Figure 6). The 5-, 7-, and 10-year ROC curves of risk score were plotted, with the AUCs of 0.684, 0.622, and 0.711, respectively (Figure 6E).

**Figure 7:**
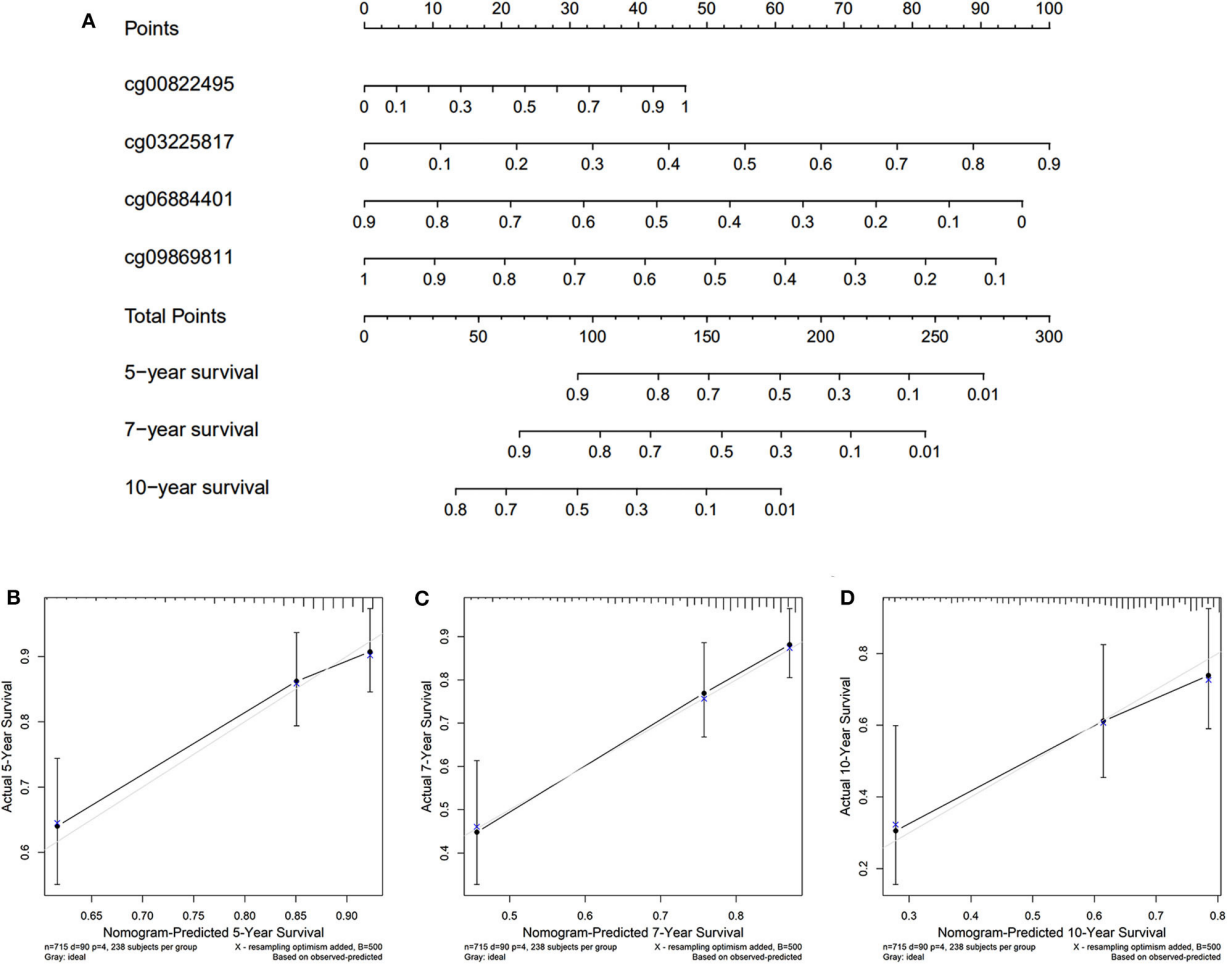
Nomogram of the TCGA cohort. **(A)** The nomogram of 5-, 7-, and 10-year OS. **(B–D)** The calibration curves of 5-, 7-, and 10-year OS of TCGA cohort. The blue dot and light gray line represent the ideal prediction model, and the black solid line represents the observed model.

The nomogram was plotted for the TCGA cohort to calculate the risk score and predict 5-, 7-, and 10-year OS of BC patients (Figure 7A). The calibration curves were also provided (Figures 7B–D). The observed model presented with black solid line seemed close to ideal prediction model presented with blue dot and light gray line. The dynamic nomogram was released online at the following website: https://cpgsignature-survival-breastcancer.shinyapps.io/cpgsignature-survival-breastcancer/.

### Analysis of Included CpG Sites

**Figure 8:**
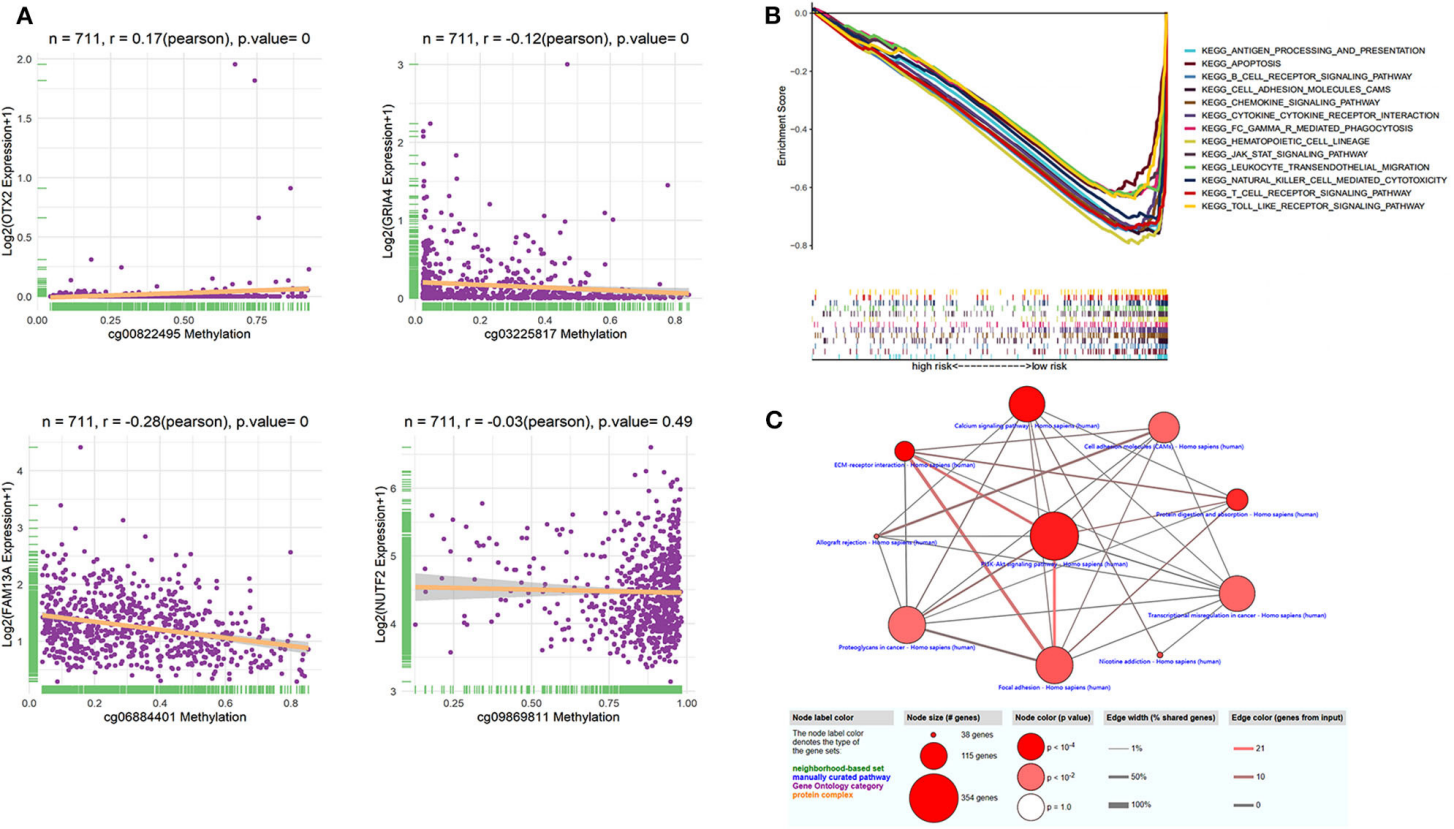
Function analysis of dmCpG-based prognostic signature. **(A)** Correlation between methylation level of dmCpG sites and corresponding gene expression. *X*-axis indicates correlation of methylation, and *Y*-axis indicates gene expression. Correlation coefficients and hypothesis tests were obtained based on Pearson correlation test. **(B)** GSEA enrichment for high- and low-risk patients classified with risk score. **(C)** KEGG enrichment of DMRs. Node indicates KEGG terms, and lines indicate interactions. Node size indicates the number of involved genes; node color indicates *P* value, edge width indicates percentage of shared genes, and edge color indicates the number of inputted genes.

The identified dmCpG sites were analyzed for further exploring their function mechanism. First, the characteristics of involved dmCpG sites were extracted, including located gene and regions (Table 2). Second, RNA-Seq data were obtained in 711 samples from TCGA cohort. The correlations between the four dmCpG sites in the signature and their target gene expression were analyzed (Figure 8A). Positive association was observed in cg00822495-OTX2 (*r* = 0.17, *P* = 0.000), whereas negative association was observed between cg06884401-FAM13A (*r* = −0.28, *P* = 0.000) and cg03225817-GRIA4 (*r* = −0.12, *P* = 0.000). No significant correlation was observed in cg09869811-NUTF2 (*r* = −0.03, *P* = 0.49) (Figure 8A). Third, GSEA was performed to identify the more active pathways in low-risk patients. Thirteen pathways were more obviously enriched, which were antigen processing and presentation, apoptosis, B-cell receptor signaling, cell adhesion molecules cams, chemokine signaling pathway, cytokine-cytokine receptor interaction, FC gamma r–mediated phagocytosis, hematopoietic cell lineage, JAK-STAT signaling pathway, leukocyte transendothelial migration, natural killer cell–mediated cytotoxicity, T-cell receptor signaling pathway, and toll-like receptor signaling pathway (Figure 8B). Finally, DMRs were explored to further understand the function mechanism of methylation. A total of 1,555 screened DMRs were annotated, and the KEGG pathway enrichment analysis was performed. The 10 most enriched pathways were provided (Figure 8C). In these pathways, PI3K-Akt signaling pathway showed most involved DMRs and frequent interactions with other terms.

**Table 2:** The characteristics of dmCpG sites.

**CpG ID**	**Gene**	**Feature**	**Ccgi**	**Feat.cgi**
cg00822495	*OTX2*	5′-UTR	island	5′-UTR-island
cg03225817	*GRIA4*	5′-UTR	island	5′-UTR-island
cg06884401	*FAM13A*	1stExon	opensea	1stExon-opensea
cg09869811	*NUTF2*	5′-UTR	opensea	5′-UTR-opensea

## Discussion

The epigenetic aberrations have been observed during the tumorigenesis and development of BC (25, 26). The DNA methylation located at gene promoter would generally suppress gene transcription, thus down-regulating the expression level of target genes. One study investigated DNA methylation with methylation quantitative trait loci of 30,477 CpG sites in 122,977 BC patients and 105,974 controls of European descent (16). They screened 199 CpG sites with significant association with BC risk. Based on the methylation detection tools and computational analysis, the relationship between DNA methylation and gene expression can be well-demonstrated for identifying new biomarkers for BC (16). Considering the benefit of early and non-invasive diagnosis, blood sample–based DNA methylation was developed. One comprehensive study identified DNA methylation markers in blood for diagnosis or risk evaluation of BC. Variations in DNA methylation profiles, both at overall genomic level and specific loci, have been associated with BC risk (17). Another large scale meta-analysis identified genes consistently associated with prognosis, and their DNA methylation could indicate prognosis and clinical stratification of BC patients (27).

Some experimental evidence explored and validated the candidate DNA methylation sites. A fast and accurate methylation marker–based automated cartridge system was developed to detect BC in cells obtained by fine-needle aspiration. The panel consisted of 10 highly methylated markers, presenting an AUC over 0.9 in discriminating BC and benign breast lesions (28). Methylation signatures involving 100 or 30 CpG probes were validated to discriminate patients with or without recurrence (29). Accumulating evidences proved the correlation between global DNA methylation and BC risk, which could be modified by reproductive characteristics (15), oxidative DNA damage (14), and chemotherapeutic agents (30).

In recent years, several prognostic prediction indexes have been developed, based on the expression levels of mRNA, lncRNAs, miRNAs, and so on. One study reported a seven-gene signature for prognostic prediction and treatment guidance in triple-negative BC (TNBC), in which recurrence risk score can be calculated as follows: mRNA signature = 1.108 × *TMEM101* – 0.213 × *KRT5* – 0.315 × *ACAN* – 0.464 × *LCA5* + 0.446 × *RPP40* – 0.373 × *LAGE3* – 0.257 × *CDKL2* (31). More studies reported that the integrated lncRNA–mRNA signature provided better prognostic prediction efficacy. The developed response score involved 1 lncRNA and 2 coding genes: response score = 2.595 × *BPESC1* – 1.09 × *WDR72* – 1.428 × *GADD45A* – 0.731 (32). Another integrated mRNA–lncRNA signature was developed based on the mRNA species for FCGR1A, RSAD2, CHRDL1, and the lncRNA species for HIF1A-AS2 and AK124454, which may be applied to predict tumor recurrence and the benefit of taxane chemotherapy in TNBC (33). A study was performed to construct mRNA-only signature and integrated mRNA–lncRNA signatures. Both signatures provided similar results for prognostic prediction, while integrated signature had a higher hazard of recurrence (34). Another systematic analysis of lncRNA–miRNA–mRNA competing endogenous RNA network identified four-lncRNA signature as a prognostic biomarker for BC, which were *ADAMTS9-AS1, LINC00536, AL391421.1*, and *LINC00491* (35).

DNA methylation was investigated as novel epigenetic biomarkers for prognostic prediction in BC, because they can increase cancer risk through altering gene expression (36). A mortality risk score based on 10 selected CpGs exhibited strong association with all-cause mortality, cardiovascular diseases, and cancer mortality in BC (37). In our study, based on the *in silico* analysis, OS-associated promoter CpG sites signature was constructed for prognostic prediction of BC, of which four dmCpG sites were identified, including cg00822495 (*OTX2*), cg03225817 (*GRIA4*), cg06884401 (*FAM13A*), and cg09869811 (*NUTF2*). In this signature, the higher methylation level of cg00822495 (*OTX2*) and cg03225817 (*GRIA4*) indicated higher risk of poor survival, while the higher methylation level of cg06884401 (*FAM13A*) and cg09869811 (*NUTF2*) indicated lower risk. Some of the involved target genes were previously reported. *OTX2* has been considered as a significantly hypermethylated gene in BC (38). *GRIA4* was a methylation-dependent gene involved in neural signaling (39). It was known as an early event in colorectal cancer carcinogenesis (40). One study has reported that rs1059122 (*FAM13A*) might result in BC susceptibility in Chinese population (41). *FAM13A* was also reported as a hypoxia-induced gene in non–small cell lung cancer, which was overexpressed under hypoxia conditions (42). *NUTF2* was a novel methylation site for BC, which has not been previously reported.

Although the CpG-based signature explored in our study has a better performance than age and TNM stage, there were several limitations to be considered. First, it was only an *in silico* and retrospective study of publicly available data. The validation of the prediction was performed in only one independent cohort. Adequate validation in a larger population-based prospective cohort should be further performed to strengthen the clinical utility. Second, the integration of risk score with other previously reported markers such as age and TNM might enable the development of more reliable biomarkers. Further study would be necessary. Third, the biological functions of some related target genes should be explored and verified.

In recent years, with the rapid development of genome-detecting technology, we are entering an era of precise treatment. A lot of biomarkers based on gene expression profiles were identified, but very few were learned from CpG dinucleotide sites. The four-CpG signature and nomogram explored in our study could guide clinicians to predict long-term survival of BC patients, accurately identify high-risk patients, and take early intervention in the treatment. It is a fact that the detection of CpG sites is more complex and expensive than gene expression detection now, but hundreds of thousands of CpG sites contain promising diagnostic and prognostic value, which would be explored with the development of detection technology in the future.

## Conclusions

This study explored a novel promoter CpG-based signature that exhibited good prognostic prediction efficacy in the long-term OS of BC patients.

## Data Availability Statement

All datasets generated for this study are included in the article/Supplementary Material.

## Author Contributions

YG analyzed the data and wrote the manuscript. XM and ZQ helped to collect relevant papers for this research. BC analyzed the data and generated the figures and tables. FJ guided the research process. All authors read and approved the final manuscript.

## Conflict of Interest

The authors declare that the research was conducted in the absence of any commercial or financial relationships that could be construed as a potential conflict of interest.
