# Supplementary material for: A Novel Promoter CpG-Based Signature for Long-Term Survival Prediction of Breast Cancer Patients
Source: Front Oncol. 2020 Oct 20;10:579692. doi: 10.3389/fonc.2020.579692 (PMC7606941; doi:10.3389/fonc.2020.579692)
Supplement: Supplementary file 1 [file Table_1.DOCX]

Supplementary materials for **“A novel promoter CpG-based signature for long-term survival prediction of breast cancer patients”**

**
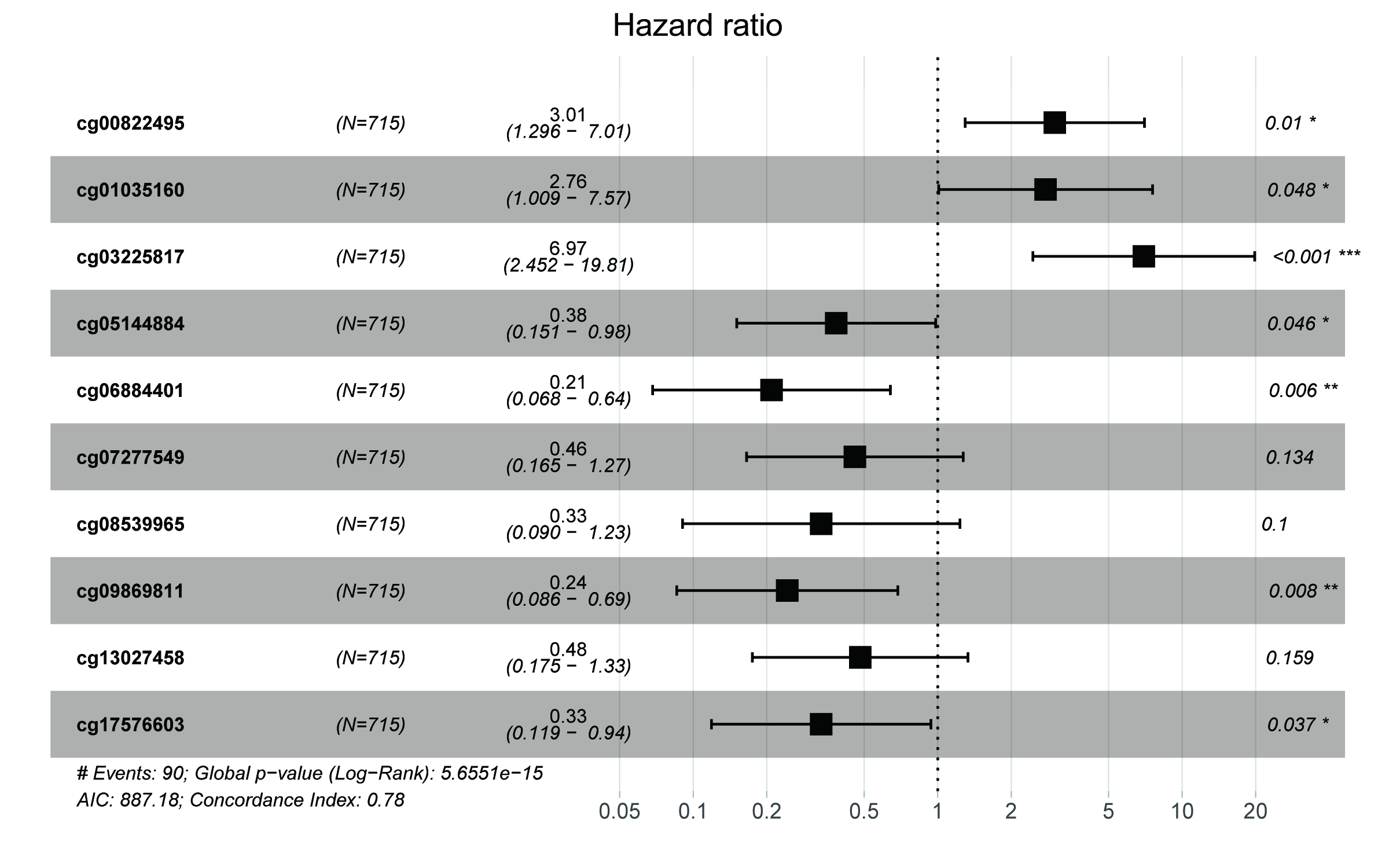
**

****Supplementary Figure S1.** The ten candidate dmCpGs sites screened with stepwise regression analysis.**
